# Cytokine Research in Depression: Principles, Challenges, and Open Questions

**DOI:** 10.3389/fpsyt.2019.00030

**Published:** 2019-02-07

**Authors:** Hubertus Himmerich, Olivia Patsalos, Nicole Lichtblau, Mohammad A. A. Ibrahim, Bethan Dalton

**Affiliations:** ^1^Department of Psychological Medicine, Institute of Psychiatry, Psychology & Neuroscience, King's College London, London, United Kingdom; ^2^South London and Maudsley NHS Foundation Trust, London, United Kingdom; ^3^Maidstone and Tunbridge Wells NHS Trust, Maidstone, United Kingdom; ^4^Department of Clinical Immunological Medicine and Allergy, King's Health Partners, King's College Hospital, London, United Kingdom

**Keywords:** cytokine, interleukin, interferon, tumor necrosis factor, depression

## Abstract

Cytokines have been implicated in the pathology of depression. Currently, the evidence is based on cross-sectional studies and meta-analytic research comparing blood concentrations of T helper type 1 (T_H_1), T helper type 2 (T_H_2), pro-inflammatory or anti-inflammatory cytokines of patients with a depressive disorder to those of healthy controls. Additionally, multiple longitudinal studies have investigated cytokine levels during antidepressant treatment. According to the current literature, it seems that peripheral levels of interleukin (IL)-6, IL-10, IL-12, IL-13, and tumor necrosis factor (TNF)-α are elevated and that interferon (IFN)-γ levels are lower in patients with depression compared to healthy controls. However, the overlap of cytokine values between acutely depressed patients, remitted and recovered patients and healthy controls is considerable. Thus, the discriminative power of cytokine concentrations between depressed and non-depressed people is likely weak. Treatment with certain antidepressants appears to decrease peripheral levels of IL-6, IL-10, and TNF-α. However, weight gain-inducing psychopharmacological substances, such as the antidepressant mirtazapine, have been reported to potentially increase the production of pro-inflammatory cytokines. Even though cytokines are often discussed as biomarkers for depression, they have also been shown to be altered in other psychiatric disorders. Moreover, many environmental, social, psychological, biological, and medical factors are also associated with cytokine changes. Thus, cytokine alterations seem extremely unspecific. The interpretation of the results of these studies remains a challenge because it is unknown which type of cells are most responsible for cytokine changes measured in the blood nor have the main target cells or target tissues been identified. The same cytokine can be produced by multiple cell types, and the same cell can produce various cytokines. Additionally, redundancy, synergy, antagonism, and signaling cascades of cytokine signaling must be considered. Cytokines might not be associated with the diagnosis of depression according to the currently used diagnostic manuals, but rather with specific subtypes of depression, or with depressive symptoms across different psychiatric diagnoses. Therefore, the currently available diagnostic systems may not be the ideal starting point for psychiatric cytokine research.

## Introduction

### Aim of This Review Article

The aim of this narrative review is to explain the fundamentals, implications, challenges, and limitations of cytokine research in depression. This comprises of:

- a brief explanation of what cytokines are,
- a short illustration of the historical developments leading to where we currently are in psychiatric cytokine research,
- an explanation of the physiological fundamentals of cytokine signaling within the immune system and the brain,
- a summary of how cytokines have been linked to depression, its risk factors and antidepressant therapy,
- a critical perspective on the limitations that researchers currently face while interpreting findings of cytokine research in depression.

Several recent meta-analyses have already summarized data on peripheral cytokine alterations in depression [e.g., (1, 2)] and during antidepressant therapy [e.g., (2)]. Therefore, this article intends to help to explain and interpret such findings in the current scientific literature. Results of animal studies, *in vitro* studies and research on serum or plasma levels of cytokines in patients with other psychiatric disorders will be mentioned, as this is necessary to understand the advantages and limitations of cytokine research in depression.

### Cytokines

The term “cytokine” is a compound word derived from the ancient Greek language. Its first component “κύτoς” means “cell,” and its second part “κíν*ησις*” means “movement.” Cytokines are a broad and loose category of secreted proteins that are important in cell signaling. This group of messenger molecules includes chemokines, interferons (IFN), interleukins (IL), lymphokines, and tumor necrosis factors (TNF). Cytokines are produced by immune cells such as macrophages, B lymphocytes, T lymphocytes, and mast cells which are mobile within the body, as well as parenchymal cells. Thus, cytokine production is not bound to a specific organ, but can happen everywhere in the human organism. Apart from immune cells, other cells that release cytokines include endothelial cells, fibroblasts, and epithelial and stromal cells within the body's periphery, and microglia and astrocytes in the brain (3–6).

Cytokines are distinct from hormones and neurotransmitters (7, 8) which are other important signaling molecules in the body. Hormones are measured in less variable concentrations in the blood circulation and are usually produced and secreted by specific cells within endocrine glands. Neurotransmitters generally transmit signals across a chemical synapse, such as a neuromuscular junction, from one nerve cell to another, or from a nerve cell to gland cell (9). Most cytokines act in their immediate micro-environment. A few exert a hormone-like effect by being released in to the blood to act on distant organs, e.g., the cytokine mediators of the acute phase response, IL-1, IL-6, and TNF-α (10).

For further information about the differences and similarities between cytokines, hormones and neurotransmitters see Table 1.

**Table 1:** Synopsis of similarities and differences in characteristics of cytokines, hormones and neurotransmitters (7–9, 11).

	**Cytokines**	**Hormones**	**Neurotransmitters**
Chemical characteristics	• Secreted proteins	• Derivatives of cholesterol • Derivatives of amino acids • Peptides • Proteins	• Gasotransmitters • Amino acids • Monoamines and trace amines • Peptides • Purines • Fatty acids • Acetylcholine
Cells of origin	• Immune cells • Endothelial & epithelial cells • Fibroblasts • Stromal cells • Microglia, Astrocytes	• Cells of endocrine glands	• Nerve cells
Target cells	• Immune cells • Nerve cells • Principally all somatic cells	• Cells of distant target organs • Principally all somatic cells	• Nerve cells • Muscle cells • Gland cells
Prototypical signaling way	• Autocrine • Paracrine • Endocrine	• Endocrine	• Synaptic
Concentration in the circulation	• Picomolar • Increase up to 1,000 times during trauma or infection	• Nanomolar • Little variation	• Spillover of neurotransmitters into the circulation only under certain circumstances
Receptors	• Transmembrane receptors linked to – JAK-STAT pathway – G-proteins – NFκB	• Transmembrane receptors linked to – G-proteins – Enzymes • Intracellular receptors linked to – DNA promotors	• Transmembrane ionotropic receptors: – Neurotransmitter-gated ion channel • Transmembrane metabotropic receptors linked to – G-proteins

### Historical Background of the Discovery of Cytokines and Their Importance for the Brain

From the 1950s to the 1980s, the first cytokines and their functions were discovered. Among those important cytokines were IFN-α (12), IFN-γ (13), the macrophage migration inhibitory factor (MIF) (14, 15), and TNF-α (16). Shortly after the discovery of the first cytokine, IFN-α, it became clear that cytokines from the body's periphery can influence inflammatory processes in the brain (17, 18), that cytokines can be produced in the brain (18), and that immune cells are not the only cells that release cytokines (19). In addition to their role in steering the immune system to defend the body from pathogens (12) and tumors (16), their modifying effect on neurotransmission was discovered (20, 21). In the 1970s, scientists started to understand that cytokines act via specific cytokine receptors on the surface of cells (22).

The first chemical analyses to measure cytokines were complicated by the low concentrations of cytokines in serum, plasma and tissue. For example, cytokines like TNF-α usually circulate in picomolar concentrations in the serum [e.g., (23)]. In contrast, classic hormones, for example cortisol, circulate in nanomolar concentrations [e.g., (24)]. The lack of assay systems was overcome by monoclonal antibody technology and the invention of the radio-immuno-assay (RIA) (25) and the enzyme-linked immunosorbant assays (ELISA) (26, 27). The RIA and ELISA allowed a highly sensitive measurement of cytokine concentrations. Further technical advancements have led to an abundance of methods to measure cytokines including bioassays, protein microarrays, high-performance liquid chromatography (HPLC), sandwich enzyme-linked immunosorbent assay (ELISA), Meso Scale Discovery (MSD) electrochemiluminescence and bead-based multiplex immunoassays (MIA) (28).

In the 1970s and 1980s, the first recombinant DNA molecules were generated (29) which allowed molecular cloning of a gene and the development of organisms that produce a protein product on the basis of such a cloned gene. Gene cloning allowed for the production of large amounts of recombinant cytokines (30).

### Difficulties of Cytokine Research in Psychiatry

Cytokine research in psychiatry faces several difficulties, such as conflicting results and high variance of cytokine values within samples. Most of the studies measure serum or plasma cytokine levels, but it has often been discussed whether these results reflect the situation in the brain (31).

Cytokine research in affective disorders reflects these difficulties in an exemplary way. Even though most studies have found, for example, elevated levels of TNF-α in the serum or plasma of depressed patients (32, 33), such positive results have not been obtained by all studies, and heterogeneity of the results between studies is large (2). Results of cytokine research which are partly contradicting and often difficult to interpret have also been obtained in post-traumatic stress disorder (PTSD) (34–36) and eating disorders (37). In order to understand the challenges and difficulties of cytokine research in psychiatry, one has to consider several characteristics of cytokine signaling and their role within the immune system. Therefore, the next sections will explain these mechanisms.

### Methods of This Review Article

This is a narrative review; therefore, we did not apply strict selection criteria for the inclusion of certain articles. We rather selected articles based on how comprehensive, innovative, and clearly written they were and how much information they provided for an in-depth understanding and a critical debate of the topic. We included not only original research, but also reviews, book chapters and case reports. Regarding the section on cytokine alterations in depression and other psychiatric disorders, we strictly included only the latest meta-analysis on cytokine levels for each diagnosis in the text and in Table 2.

**Table 2:** Summary of cytokine blood concentrations in the context of psychiatric disorders according to relevant meta-analyses (2, 36–40).

**Diagnoses**	**Cytokines**										**References**
	**IL-1β**	**IL-2**	**IL-4**	**IL-6**	**IL-8**	**IL-10**	**IL-12**	**IFN-γ**	**TNF-α**	**TGF-β**	
**AFFECTIVE DISORDERS**											
Depression	↔	↔	↔	↑	↔	↑	↑	↓	↑	↔	Köhler et al. (2)
BAD	↑	↔	↑	↔	↔	↑		↔	↑		Modabbernia et al. (39)
*- Manic*				↑		↔			↑		Goldsmith et al. (38)
*- Depressed*			↑	↔		↔			↔		Goldsmith et al. (38)
*- Euthymic*		↑				↑		↑	↑		Goldsmith et al. (38)
**SCHIZOPHRENIA**											
First episode	↑	↔		↑	↑	↑	↑	↑		↑	Goldsmith et al. (38)
Acute relapse	↑	↔	↓	↑	↑	↓	↑	↑	↑	↑	Goldsmith et al. (38)
**TRAUMA- AND STRESSOR-RELATED DISORDERS**											
PTSD	↑	↔	↔	↑	↔	↔		↑	↔		Passos et al. (36)
**OBSESSIVE-COMPULSIVE DISORDERS**											
OCD	↓			↔					↔		Gray and Bloch (40)
**EATING DISORDERS**											
AN	↔			↑					↑	↔	Dalton et al. (37)
BN				↔					↔		Dalton et al. (37)

*↑, increase; ↔, no difference; ↓, decrease in cytokine levels; IL, interleukin; TNF-α, tumor necrosis factor; TGF, transforming growth factor; BAD, bipolar affective disorder; PTSD, post-traumatic stress disorder; OCD, obsessive-compulsive disorder; AN, anorexia nervosa; BN, bulimia nervosa*.

## Characteristics of cytokine signaling

### Cytokine Release

Cytokines mediate and regulate the immune system. Their secretion is brief and self-limited.

There are three main types of cytokine signaling: autocrine, paracrine and endocrine (7, 8, 41). An example for autocrine signaling is a T-helper type 2 (T_H_2) cell which can stimulate its own growth by producing IL-4 (42). The way in which T_H_2 cells can also stimulate nearby B lymphocytes by releasing IL-4 is an example of paracrine signaling (43) and endocrine signaling can be illustrated by TNF-α which is produced by macrophages in the adipose tissue and secreted into circulation. This pro-inflammatory signal can contribute to inflammatory processes within the artery walls and eventually lead to arteriosclerosis (44). In the cases of trauma, infection, stress, neoplasia, and inflammation, the body activates a complex systemic early-defense system. This process is called acute phase response which is a specialized systemic innate immune response in which the cytokines IL-1, IL-6, and TNF-α act like hormones. They are released by innate immune cells at the sites of infection or inflammation and released into circulation to act on distant organs e.g., the liver, to mediate the release of acute phase reactants, e.g., C-reactive protein (CRP) (45).

The same cytokine can be produced by multiple cell types. For instance, TNF-α is released by white blood cells, the endothelium, fat cells and other cells (46). Furthermore, one single cell can produce different cytokines. For example, T_H_2 cells can produce IL-3, IL-4, IL-5, IL-6, and IL-13 (47).

### Cytokine Effects

One cytokine can act on multiple cell types and have different effects on these cells (48). For example, IL-4 can induce activation of B lymphocytes but inhibit T helper type 1 (T_H_1) cells (49). It can further lead to differentiation of cytotoxic T cell precursors and proliferation in mast cells (50). Regarding TNF-α, all cell types of the body express TNF-α receptor type 1 (TNF-R1) (51). When bound to TNF-α, these TNF receptors transduce growth regulatory signals. TNF-α is able to initiate apoptosis in some cells causing DNA fragmentation and cytolysis, but also cell growth and differentiation in others. Whether TNF-α induces cell differentiation or apoptosis depends on the signaling pathway activated within the cell. The NF-κB signaling pathway will lead to cell differentiation, whereas cells in which caspases are activated are more likely to undergo apoptosis in response to TNF-α (24). IL-10 can be inhibitory to macrophages and T_H_1 cells, yet activating for T_H_2 cells and B cells and can thus be immunosuppressive as well as immunostimulatory (52). The phenomenon that one cytokine can have diverse effects on different cells is called pleiotropy.

Cytokine signaling shows redundancy, because two or more cytokines can have a similar function. IL-2 and IL-4 both enhance T cell proliferation (53). All three IFNs, IFN-α, IFN-β, and IFN-γ, increase the activity of natural killer lymphocytes and stimulate the synthesis of arachidonic acid products (54). IFN-γ, IL-2, and TNF-α promote cellular immunity and the activation of cytotoxic cell contacts (55).

Another typical phenomenon in cytokine signaling is synergy which is a strong combined effect of 2 cytokines when acting together e.g., IL-3 and IL-4 amplify each other to induce growth, differentiation, and activation of mast cells in a synergistic way (47). Cytokines can also antagonize each other's effects. For example, IL-12 which activates T_H_1 cells can be blocked by IL-4 (56–58). Another example of cytokine antagonism is that cytokines of the IL-1 superfamily can antagonize IL-18 effects (59). Additionally, cytokine cascades play a significant role in cytokine signaling which means that an activation of one cytokine produced by one cell type induces cytokine production by other cell types. For example, IL-4 induces the expression of IL-3, IL-5, and IL-13 (60).

## Cytokines Within the Immune System

### The Role of Cytokines Possibly Relevant for Research in Affective Disorders Within the Immune System

Many cytokines with vital roles for the regulation of the immune system have been investigated in affective disorders. Examples of such immunologically important cytokines measured in the serum of patients with depression are IL-1, IL-2, IL-4, IL-5, IL-6, IL-10, IL-12, IL-13, IL-17, IFN-α, IFN-γ, TNF-α, and transforming growth factor (TGF)-β (2, 33, 61–64). Other cytokines with immunologically important functions are IL-21 (65) and IL-22 (66), though both of which have only recently gained the interest of the depression research field. IL-21 has been studied regarding its role in response to treatment with the antipsychotic aripiprazole (67), which can also be used as an augmentation strategy in treatment-resistant depression (68), and IL-22 production has been shown to increase during exposure to antidepressants like citalopram or mirtazapine (69).

**Figure 1:**
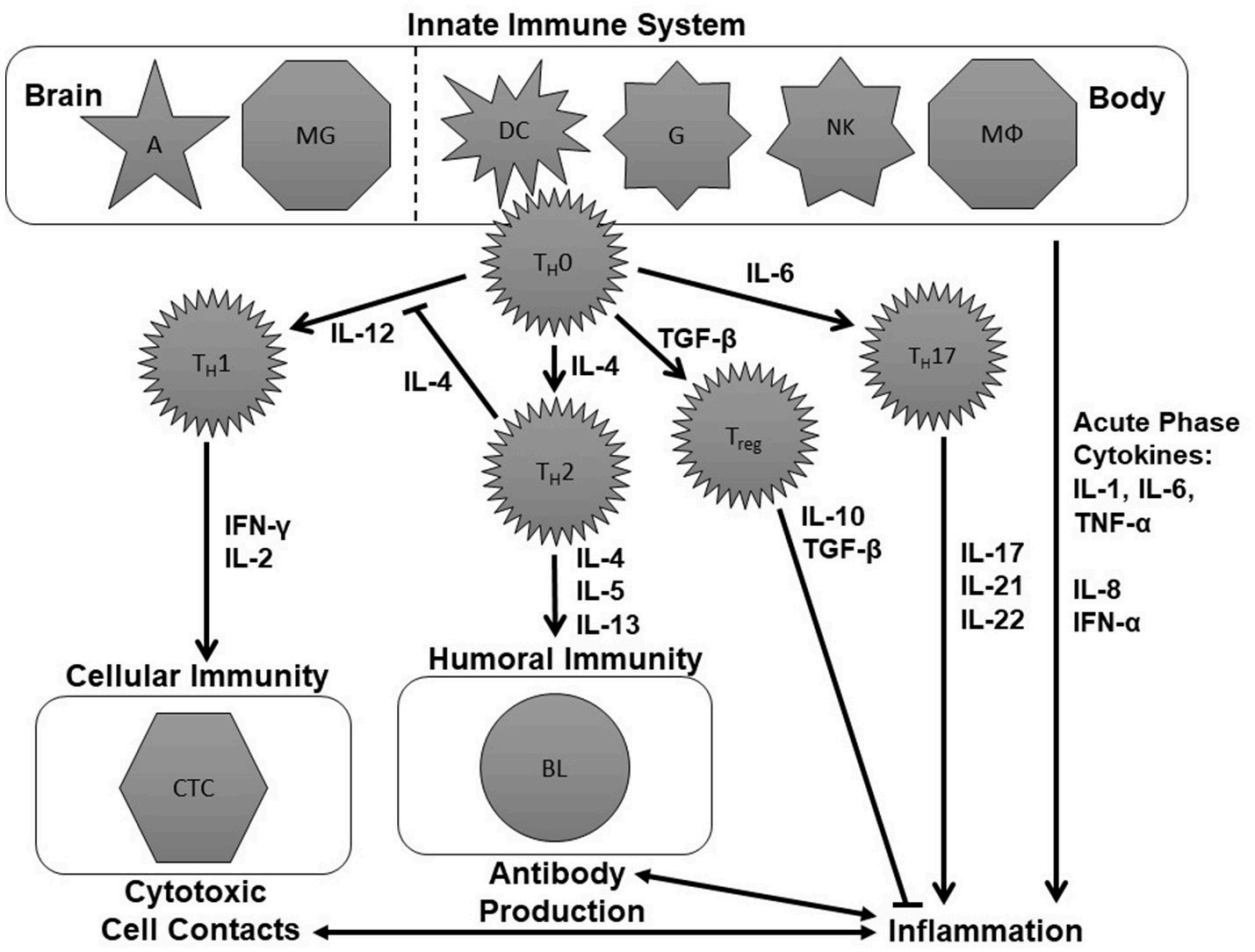
Schematic figure of the immune system. This is an over-simplified representation of the immune system, deliberately so, to focus on aspects that have already been investigated in the immune-psychiatry literature. The tasks of the innate immune system are performed by astrocytes (A), microglia (MG), dendritic cells (DC), naïve granulocytes (G), natural killer cells (NK), and macrophages (MΦ). These cells modulate the further immune response involving naïve T-helper (T_H_0), T helper 1 (T_H_1), T helper 2 (T_H_2), and T helper 17 (T_H_17) cells, regulatory T cells (T_reg_) as well as cytotoxic T cells and B lymphocytes (BL) by the production of cytokines including interleukins (IL), interferons (IFN), tumor necrosis factor-α (TNF-α), and transforming growth factor-β (TGF-β). For further details see text.

Figure 1 is an attempt to create a simplified schematic figure that depicts the role of these cytokines within the immune system. This figure tries to bring some order to the different cytokines measured in psychiatric research, which are often discussed without explaining the immunological context of these molecules.

The immune system is divided into the innate and the adaptive immune response. The innate immune system can fight pathogens without a previous contact with them. The tasks of the innate immune system are performed by astrocytes (A), microglia (MG), dendritic cells (DC), granulocytes (G), natural killer cells (NKZ), and macrophages (MΦ) (upper part of Figure 1). These cells modulate the further immune response by the production of cytokines like IL-1, IL-4, IL-6, IL-12, and TNF-α.

To understand the adaptive immune system, we will assume two different scenarios that lead either to specific cytotoxic cell contacts or the production of specific antibodies (see left middle and lower part of Figure 1). One scenario within the immune system could be a viral infection. This can lead to the production of IL-12 by cells of the innate immune system which activates T_H_1 cells. Consequently, they produce IFN-γ, IL-2, and TNF-α which, in turn, activate cytotoxic T cells. Cytotoxic T cells can destroy the virus-infected cells (70). Another potential scenario would be that cells of the innate immune system release IL-4 following a bacterial infection. IL-4 stimulates T_H_2cells. T_H_2 cells, in turn, will produce IL-4, IL-5 and IL-13 and thereby induce the production of antibodies which can help to mark and eliminate the pathogens (70).

If the cells of the innate immune system produce IL-1, IL-6 or TNF-α, the consequence is an activation of the so-called T helper 17 (T_H_17) cells which produce cytokines like IL-17, IL-21, and IL-22. Together with IL-1, IL-6, IL-8, TNF-α and IFN-α, IL-17, IL-21, and IL-22 are cytokines which promote inflammation. Therefore, they are called pro-inflammatory cytokines (71, 72). Inflammatory processes, in turn, are bidirectionally linked to cytotoxic and antibody-driven mechanisms. An anti-inflammatory effect, in contrast, occurs when naïve T helper (T_H_0) cells produce TGF-β. TGF-β activates regulatory T (T_reg_) cells which produce IL-10 and further TGF-β. These cytokines have an anti-inflammatory effect (73) (see right middle and lower part of Figure 1).

### Types of Cytokines According to Their Immunological Function

With this knowledge, we can distinguish between four categories of cytokines which are often talked about in the psychoimmunological literature:

- T_H_1 cytokines (IL-2, IL-12, IFN-γ) which fuel the T_H_1 branch of the immune system and lead to cytotoxic cell contacts.
- T_H_2 cytokines (IL-4, IL-5, IL-13) which stimulate the T_H_2 branch of the immune system and induce the production of antibodies.
- Pro-inflammatory cytokines (IL-1, IL-6, IL-8, IL-17, IL-21, IL-22, IFN-α, TNF-α) which promote inflammation.
- Anti-inflammatory cytokines (IL-10, TGF-β) which are influenced by regulatory T cells and prevent inflammatory processes from escalating.

Even though these categories are often referred to in the psychoimmunological literature, these are neither distinct nor generally accepted categories for the classification of cytokines. However, this functional classification might help to understand recent research papers on pro-inflammatory, anti-inflammatory, T_H_1 and T_H_2 cytokines in depression [e.g., (74–77)]. One must also take into account that cytokines can have different effects on different cells and thus may have pro- but also anti-inflammatory properties. Moreover, cytokines produced by certain T helper cells, for example IL-13 which is produced by T_H_2 cells, have anti-inflammatory properties (78). Thus, they can be T_H_2 as well as anti-inflammatory cytokines. Even though IFN-α has been listed in this figure as a pro-inflammatory cytokine, IFN-α also has several anti-inflammatory properties (79).

## The Influence of Cytokines on the Brain

### How Cytokines Enter the Brain

As previously mentioned, cytokines can be produced by neurons, astrocytes and microglia within the brain (see Figure 1). Additionally, peripherally produced cytokines can access and affect the brain through three pathways: humoral, neural, and cellular (31).

The humoral pathway describes when cytokines access the brain through leaky sections of the blood-brain barrier such as the choroid plexus and the circumventricular organs. The neural pathway involves the stimulation of primary afferent nerve fibers in the vagus nerve by pro-inflammatory cytokines. The cellular pathway describes how pro-inflammatory cytokines stimulate microglia to produce monocyte chemoattractant protein-1 (MCP-1), which subsequently recruits monocytes to the meninges and brain parenchyma (31, 80).

### Influence on Neurotransmitter Metabolism and Signaling

Given that peripheral production of cytokines has been associated with depression (2, 81), and that peripherally produced cytokines can influence the central nervous system (CNS), the impact of cytokines on neurotransmitters such as serotonin has been studied extensively. For example, animal (82–84) and clinical (85, 86) studies have found an altered metabolism of serotonin resulting from cytokine exposure. Cytokines can decrease serotonin synthesis by activating the enzyme indoleamine 2,3 dioxygenase (IDO) which breaks down tryptophan, the amino acid precursor of serotonin, to kynurenin (KYN) instead of metabolizing tryptophan to serotonin. This process of serotonin depletion has been postulated to be associated with major depression (87, 88). Cytokines also influence the synthesis of monoamines through disruption of tetrahydrobiopterin, an essential enzyme co-factor to hydroxylases involved in monoamine synthesis. Furthermore, they can modulate serotonin signaling by increasing the expression and function of monoamine transporters which perform the re-uptake of synaptic serotonin (89–92). Moreover, pro-inflammatory cytokines can affect the release of neurotransmitters (93). For example, glutamate release by astrocytes has been shown to be affected by cytokines, potentially leading to excitotoxicity (94, 95). In addition, pro-inflammatory cytokines have been implicated in the stimulation of N-methyl-D-aspartate (NMDA) receptors, and the inhibition of γ-aminobutyric acid (GABA) and acetylcholine signaling (96).

Furthermore, cytokines are involved in autoimmunity, including the production of autoantibodies. For example, autoantibodies against dopamine-2 receptors have been found to contribute to the development of pediatric neuropsychiatric disorders associated with streptococcal infection and subjects with Tourette's syndrome (97). Autoantibodies against serotonergic and cholinergic receptors are thought to induce depressive syndromes (98).

### Influence on Neuroendocrine Signaling

Cytokines can also affect neuroendocrine function by increasing hypothalamic-pituitary-adrenal (HPA) axis activity. Acute cytokine administration has been shown to increase corticotropin-releasing hormone (CRH), adrenocorticotropic hormone (ACTH), and cortisol release, all of which have been found to be elevated in patients with major depression (99–101). In contrast, chronic cytokine administration is associated with a flattening of the diurnal cortisol curve, which has been linked to adverse behavioral effects and poor outcomes in several illnesses such as cardiovascular disorders and cancer (102–104). Inflammatory cytokines are hypothesized to exert these effects through the disruption of the cortisol receptor's (glucocorticoid receptor) expression and function (101).

### Influence on Neurogenesis and Autoimmune Destruction of Nerve Cells

Neurogenesis is another aspect of brain activity influenced by cytokines. As mentioned previously, certain pro-inflammatory cytokines stimulate glutamate release by astrocytes. Glutamate can have a detrimental effect on neurogenesis by binding to extra-synaptic NMDA receptors, leading to a decrease in brain-derived neurotrophic factor (BDNF), which is pivotal for neurogenesis (105). Additionally, an activation of TNF-α signaling has been suggested to contribute to the destruction of hypocretin neurons in patients with narcolepsy (23, 106).

### Influence on Specific Brain Regions

Cytokines' effects on specific brain regions have been demonstrated through imaging techniques. Among those brain regions influenced by cytokines are the basal ganglia, which are involved in motor activity and motivation, the dorsal anterior cingulate cortex (ACC), which is a central area for the generation of anxiety, and the subgenual ACC, which has been reported to be involved in the development of depression (107, 108).

Positron emission tomography (PET) studies have revealed that the application of IFN-α leads to increases in basal ganglia glucose metabolism (109, 110). This is of specific interest for cytokine research in depression because depression and fatigue are the main side effects of treatment with IFN-α (111, 112). IFN-α has also been implicated in increased ACC activation as evidenced by fMRI studies (113, 114), and such ACC activation is associated with increased anxiety, arousal, obsessive-compulsive disorder and bipolar affective disorder (108).

## Cytokine Alterations in Depression and Other Psychiatric Disorders

### Cytokine Alterations in Patients With Affective Disorders

Regarding the main cytokines examined in affective disorders, meta-analytic research has revealed that patients with depression have elevated serum or plasma concentrations of IL-6, IL-10, IL-12, IL-13, IL-18, TNF-α, and its receptor soluble TNFR2 compared to healthy controls, whereas IFN-γ levels are lower in patients with depression than healthy controls (2, 33, 61–63). Similarly, production of IL-6, IL-10, and TNF-α levels have been shown to be increased in animal models for depression using acute or restraint stress, whereas IFN-γ production was significantly decreased by restraint stress in rats (115). In bipolar disorder, Munkholm et al. (116) found significantly higher serum or plasma levels of TNF-α, its receptor soluble TNFR1 and IL-4. However, these cytokines have not been found to be elevated by all studies in patients with depression and bipolar patients (2, 116).

### Cytokine Alterations in Patients With Psychiatric Disorders in General

The aforementioned cytokine alterations in affective disorders are not specific to major depression or bipolar disorder. For example, elevated levels of TNF-α have also been found in PTSD studies (34), in patients with anorexia nervosa (37) and in patients with acute relapse of schizophrenia (31, 38). An overview of meta-analytic studies on cytokine research in selected psychiatric disorders is shown in Table 2.

If we take a closer look at the raw data of specific studies, we see high variation in cytokine levels within groups of patients or healthy controls. For example, in a paper published by Schmidt et al. (33), mean TNF-α serum concentrations of depressed patients were 50.35 pg/ml with a standard deviation of 78.01 pg/ml, whereas mean TNF-α levels of healthy controls were 33.80 ± 46.17 pg/ml. This range indicates that, despite the significant difference between the means of both groups, depressed participants and healthy controls did not have distinct TNF-α levels, but rather overlapping ranges of TNF-α levels.

## Possible Reasons for Cytokine Alterations

### Direction of Causality

Even though there is considerable evidence for an involvement of cytokines in the pathophysiology of many psychiatric disorders, the directionality of this relationship has not yet been elucidated with certainty. However, evidence is increasing that inflammation and specifically cytokine signaling play a role in the pathophysiology of psychiatric disorders. Such evidence derives from genetic studies, long-term cohort studies and from studies investigating people with inflammatory diseases. Nevertheless, cytokine changes might also be the consequence of a psychiatric disorder, for example they can be a consequence of treatment with psychopharmacological agents or of weight changes that appear during acute episodes of the disorder or during recovery (117).

There is considerable evidence that genetic risk factors for psychiatric disorders are closely related to cytokines or other functions of the immune system. Genome-wide association studies (GWAS) have identified immune pathway genes that significantly contribute to the risk of psychiatric disorders (118). Psychiatrically relevant genes include, for example, those in the human leukocyte antigen (HLA) gene complex or rare copy number variants important for immune function (119).

The results of the Whitehall II cohort study in which British civil workers were monitored for CRP, IL-6 and cognitive symptoms of depression from 1991 to 2004 (baseline and follow-up) suggest that the inflammatory markers measured predicted symptoms of depression at follow-up, but not the other way around (120).

Studies have shown that CNS inflammation caused by infection can lead to the development of psychiatric symptoms (121) or full-blown syndromes such as depressive or manic episodes (122). However, it must be mentioned that the observation that inflammatory diseases can lead to psychiatric disorders, such as depression, is not an achievement of recent decades of research: a meta-analytic scientific approach to such observations had already been published by Emil Kraepelin in 1881 and 1882 (123).

### Risk Factors for Psychiatric Disorders Associated With Changes in Cytokine Production

If we consider the risk factors of depressive disorder, we will find familial and developmental risk factors, factors related to the natural and social environment, psychological and medical risk factors, as well as molecular factors related to genetics, epigenetics, gene expression and the brain, and the endocrine and the immune system (124–126). Table 3 provides evidence from the scientific literature that all of these risk factors for depression have been shown or suggested to be associated with alterations in cytokine production or cytokine signaling. Thus, the cytokine system seems to be involved in almost all possible predisposing or precipitating risk factors for depression that contribute to the presentation of the disorder. A risk factor like acute or chronic stress could, for example, lead to an increased production of pro-inflammatory cytokines (115, 138–140), and these cytokines could then enter the brain (31) and lead to changes in neurotransmitter systems involved in the development of depression, such as the serotonin system (82, 83, 85, 86, 182).

**Table 3:** Possible risk factors for the development of depression which have also been linked to alterations in cytokine production and signaling.

**Adolescent age which goes along with**
– Neurodevelopmental changes (127) – Hormonal changes (127)
**Environmental factors**
– Air pollution (128, 129)
**Social risk factors**
– Poverty (129) – Low socioeconomic status disadvantage (130) – Unemployment (131)
**Oxidative stress** (132)
**Nutrition and the gut microbiome**
– Vitamin deficiency, e.g., vitamin D deficiency (133, 134) – ω3/ω6 fatty acids ratio (135) – Changes in the gut microbiome (136, 137)
**Psychological risk factors**
– Poor attachment in childhood and adolescence (130) – Stress (138–140) – Bereavement (141) – Loneliness (142) – Psychological Trauma or PTSD (34, 35, 129) – Critical life events – Predisposing temperament and personality traits (143–145) – Subclinical depression (142)
**Family history of depression** (130)
**Psychiatric disorders**
– Anxiety disorders (146, 147) – Substance abuse disorders (148, 149) – Post-traumatic stress disorder (34–36)
**Brain diseases**
– Stroke (150) – Epilepsy (151, 152) – Parkinson's disease (153, 154) – Multiple Sclerosis (155)
**Physical diseases**
– Infectious diseases (62, 156, 157) – Autoimmune diseases (158, 159) – Endocrine and hormonal diseases (160) – Cancer (161) – Obesity (162, 163) – Diabetes (164) – Myocardial infarction (165) – Physical trauma (166)
**Therapies for physical diseases**
– Medications (167) – Chemotherapy (168) – Surgery (169) – Transplantation (170)
**Disturbed sleep-wake-rhythm**
– Disturbed sleep (171) – Disturbed wakefulness (172)
**Hormonal changes**
– Pregnancy (173) – Birth (174) – Menopause (175)
**Functional and structural changes in brain areas:**
– Hippocampus (128, 176) – Amygdala (177) – Prefrontal cortex (178)
**Genetic risk factors**
– Susceptibility and candidate genes (179) – Gene expression (140) – Epigenetics (180)
**Neurochemical risk factors in**
– Neurotransmitter systems (31, 81, 88, 91) – Endocrine systems (99–101) – Immune system (181)

*The cited literature refers to articles that report, review, or hypothesize an association of the risk factor in question with changes in cytokine production or cytokine signaling*.

### Confounding Factors

There are a number of confounding factors that influence cytokine serum or plasma levels. Important confounders include aging, body weight, smoking, excessive alcohol consumption, and medication (183).

Normal aging is marked by chronic low-level inflammation characterized by over-expression of circulating pro-inflammatory factors (184, 185). Chronic inflammation with increased circulating levels of cytokines is also characteristic of obesity (162, 186, 187). Adipose tissue is known to accumulate and activate macrophages and lymphocytes which secrete inflammatory factors (186, 187). Both the obese and the elderly exhibit behavioral symptoms such as depression and cognitive dysfunction at an increased rate compared to the general population. Several studies have shown that the elevated levels of inflammation may contribute to the prevalence of neuropsychiatric disorders in these populations (188–192).

Smoking (193), excessive alcohol consumption (148), and drug abuse (149) have been associated with inflammatory changes in the immune system, as reflected by an increase of pro-inflammatory cytokines. Also, psychopharmacological medication, specifically those agents which lead to weight gain, have been reported to activate cytokine production (117, 194). It is not clear, however, whether the activation of cytokines by psychotropic drugs is the cause or a consequence of weight gain in the course of psychopharmacological treatment. As growing white adipose tissue becomes infiltrated by macrophages, this fatty tissue could be a major source of pro-inflammatory cytokines in the context of increasing body weight during psychopharmacological treatment (195).

One has to keep in mind, however, that alcohol misuse (148), obesity (163) are not only confounding factors, but also risk factors for the development of depression. Therefore, they should not be dismissed as mere confounders whilst performing research on causative factors of affective disorders.

### Physical Disorders and Their Therapy

Many physical disorders have been reported to increase the risk for developing a depressive disorder (125) and to activate the production of pro-inflammatory cytokines. Examples are autoimmune and infectious diseases (156–159), endocrine and hormonal diseases (160), cancer (161), diabetes (164), myocardial infarction (165, 196), and physical trauma (166). The treatment of physical disorders, e.g., interferon-based or virostatic treatments for hepatitis C (167, 197), chemotherapy for the treatment of cancer (168), surgery (169) or transplantation (170), can lead to additional cytokine release.

### Food and the Microbiome

Vitamin deficiency, e.g., vitamin D deficiency, has also been shown to increase pro-inflammatory cytokine production and to lead to depressive symptoms (133, 134). Another example of how food can influence the immune system is the anti-inflammatory effect of ω3 fatty acids, which can reduce the production of IL-1β, IL-6, and TNF-α. ω6 fatty acids, in contrast, promote the production of these pro-inflammatory cytokines. Therefore, foods with a high ratio of ω3/ω6 fatty acids can dampen inflammatory processes and thus potentially prevent or ameliorate depressive symptoms (135).

There is an increasing body of evidence for an influence of the gut microbiome on cytokine signaling and on the development of depression. Gut microbes have, for example, been found to be capable of producing certain neurotransmitters and of influencing the central neurochemistry of the brain and thus human behavior (136). Molecules produced by microbiota, e.g. LPS, can induce cytokine production (198), and lead to depressive symptoms (199). The microbiome is linked to food intake and diet. However, the relationship is complex (200).

## Cytokine Changes During Treatment for Depression

### The Influence of Antidepressants on Cytokine Production

Some studies have investigated whether cytokine serum or plasma concentrations *in vivo* change during treatment with antidepressants. However, the results are conflicting. For example, in a study by Kraus et al. (201), TNF-α levels were measured longitudinally during treatment with mirtazapine or venlafaxine. Whereas, mirtazapine induced a significant increase in the plasma levels of TNF-α and both soluble TNF receptors, venlafaxine did not alter plasma levels of TNF-α, or soluble TNF receptors significantly (201). These findings that mirtazapine increases circulating TNF-α levels were supported by Kast et al. (202). In contrast, however, Gupta et al. (203) found that successful treatment with mirtazapine led to a decrease in serum TNF-α levels. There is currently not enough scientific literature available to draw firm conclusions about the influence of certain antidepressants on plasma or serum levels of cytokines *in vivo*. However, a recent meta-analysis which included data derived from 45 longitudinal studies and more than 1,500 patients found that antidepressant treatment, overall, decreases peripheral levels of IL-6, IL-10, and TNF-α (2).

Certain subgroups of depressed patients, for example those with psychotic depression, are usually treated with a combination of an antidepressant and an antipsychotic drug (204). Among antipsychotics, it has been shown that those with the highest risk of weight gain, for example clozapine and olanzapine (117), lead to a significant increase in pro-inflammatory cytokine levels in the blood (194). For patients with recurrent episodes of depression or bipolar depression, the treatment with mood stabilizers is recommended (205). Some of these mood stabilizers, for example lithium and carbamazepine, have also been shown to lead to weight gain as well as an increase in pro-inflammatory cytokine levels (206).

The *in vitro* literature on antidepressants suggest that some antidepressants, such as clomipramine and fluoxetine, decrease IL-6, IFN-γ, and TNF-α, whilst others like mirtazapine and venlafaxine tend to increase their levels (207). From these results, one is tempted to draw the conclusion that serotonin reuptake inhibitors (SSRIs) or serotonin and noradrenalin reuptake inhibitors (SNRI) generally decrease IL-6, IFN-γ, and TNF-α levels. However, the SSRI citalopram increased the production of IL-1β, IL-6, and TNF-α in another *in vitro* study (69). What *in vitro* studies clearly show, however, is that antidepressants (69), antipsychotics (208) and mood stabilizers (209) have a direct influence on cytokine production within the blood.

### Cytokine Levels and Antidepressant Response

Occasionally, studies have reported that baseline levels of certain cytokines or cytokine changes during treatment were associated with antidepressant treatment response during treatment with specific antidepressants or a certain combination of antidepressants. For example, Jha et al. (210) found that higher baseline levels of IL-17 were associated with greater symptomatic reduction in depressed patients treated with a bupropion-SSRI combination. However, the research in this area is sparse, and therefore, it is too early to draw far reaching conclusions from such observations. Regarding changes of cytokine levels during antidepressant treatment, the aforementioned recent meta-analysis of Köhler et al. (2) did not provide evidence that reductions in peripheral inflammation are associated with antidepressant treatment response.

### Cytokine Levels and Psychotherapy

Not only antidepressants, but also psychotherapy has been reported to be associated with cytokine changes. For example, Del Grande da Silva et al. (211) reported a clinical study showing that successful brief psychodynamic psychotherapy leads to a reduction of pro-inflammatory cytokine serum levels.

## Discussion

### Historical Considerations

The close relationship between inflammatory processes and psychiatric symptoms has been scientifically investigated since the 19th century (123). Shortly after the discovery of the first cytokine, IFN-α (12), it became clear that this cytokine was able to influence immunological processes in the brain even when peripherally administered (17, 18) and that it can be produced by cells within the brain (18). Therefore, even though cytokines were discovered as messenger molecules with important immunological functions, it quickly became clear that they also play an important role within and for the brain.

### Difficulties in Interpreting the Results of Cytokine Research

During the 1970s it became clear that immune cells are not the only cells that release cytokines (19). For example, TNF-α can be produced by white blood cells, the endothelium, fat cells and other cells (46). Therefore, if we measure a specific cytokine like TNF-α in the serum or plasma, it is unclear from which cells or which organ it is derived and where this cytokine will exert its effect, as one cytokine can have different effects on different cell types (10).

As cytokine signaling often shows redundancy (53–55), synergy (47), antagonistic effects (56–59) or signaling cascades (60), it seems advisable to take all cytokines that work together or against each other into account and thus measure a whole range instead of a single cytokine.

### Cytokines as Potential Biomarkers of Depression

As peripheral levels of IL-6, IL-10, IL-12, IL13, and TNF-α have been shown to be significantly elevated and IFN-γ plasma concentrations significantly lower in patients with depression compared to healthy controls in meta-analytic research (2), one could think that cytokines qualify as a biomarker of depression. This perspective could be supported by further meta-analytic findings that antidepressant treatment decreases peripheral levels of IL-6, IL-10, and TNF-α (2). However, one has to take into account that these are results comparing means of groups. There is huge overlap of the distributions of cytokine levels of depressed patients and healthy controls, and additionally, the meta-analytic research of Köhler et al. (2) did not provide evidence that reductions in peripheral inflammation are associated with antidepressant treatment response. Thus, the mentioned group effects may not be relevant on an individual level. Additionally, the described effects of antidepressants on certain cytokines may be a mere pharmacological effect of these medications on immune cells that is not necessarily related to the depressive syndrome, as it has been shown that antidepressants (69), antipsychotics (208), and mood stabilizers (209) influence cytokine production directly.

### Challenges in Regard to the Current Diagnostic Criteria

People with depression exhibit heterogeneous sets of symptoms. Thus, it may be sensible to define subgroups of patients with depression with more homogenous psychopathology. It might well be that within such a subgroup, cytokines are of greater value as an individualized biomarker of disease severity and antidepressant response. However, an alternative perspective may also be worth considering: cytokines could be associated with depressive syndromes independent of the psychiatric diagnosis. For example, patients with schizophrenia can also suffer from depressive symptoms (212). Therefore, cytokine levels may not only be associated with a diagnostic category, but with transdiagnostic depressive symptoms. This might be a reason for limited specificity of cytokines as biomarkers for a certain psychiatric diagnosis, because the mentioned cytokine levels are not only elevated in depression, but also in other psychiatric disorders (see Table 2). Moreover, a number of environmental, social, psychological and medical factors (see Table 3) are also associated with cytokine changes. Therefore, cytokines may currently not be considered as specific biomarkers for depression.

### Future Perspectives on Cytokines as Biomarkers for Depression

There are many immunologically important cytokines like IL-21 (65) and IL-22 (66) which have not been extensively researched in psychiatric samples yet, even though the first preliminary studies have revealed promising results (67, 69). Cytokine levels are often researched and interpreted in isolation from their origin in terms of the specific cell type and the particular tissue they are originating from, even though changes in specific cytokine-producing immune cells like T_reg_ cells have been reported in depression (213) and during antidepressant therapy (214). Additionally, their receptors and their target tissue have been neglected in psychiatric research, despite considerations that modulation of cytokine receptors might be a promising future antidepressant strategy (215).

At this point, we would like to mention that there are only few studies available that measured cytokine concentrations in the cerebrospinal fluid [e.g., (216)], even though one would assume that cytokine concentrations in the cerebrospinal fluid might better reflect cytokine signaling in the brain than cytokine levels in the serum or plasma.

Taken together, including novel cytokines, cells and tissues of their origin, their receptors and target tissues in future scientific and clinical projects might help to fill the gaps in our knowledge in immunological biomarker research.

### Future Perspectives on Cytokines as a Therapeutic Target for Antidepressant Treatment

Animal experiments have shown that cytokine blockers like TNF-α blockers can be effective in the treatment of depression-like behavior (217). However, attempts to try cytokine blockers in people with depression did not show striking success (218). Furthermore, designer monoclonal antibodies to bind directly to the cytokine and soluble cytokine receptors are currently being developed which will hopefully have less severe side effects than those currently available.

## Author Contributions

HH, OP, NL, and BD conducted the literature search. HH and MI drafted the manuscript. All authors were involved in drafting, critiquing and approving of the manuscript, and accept responsibility for the accuracy, and integrity of this work. The authors were the only individuals who contributed to this publication.

### Conflict of Interest Statement

The authors declare that the research was conducted in the absence of any commercial or financial relationships that could be construed as a potential conflict of interest.
